# Automated Detection of Seizure Types from the Higher-Order Moments of Maximal Overlap Wavelet Distribution

**DOI:** 10.3390/diagnostics13040621

**Published:** 2023-02-08

**Authors:** Joseph Mathew, Natarajan Sivakumaran, P. A. Karthick

**Affiliations:** 1 Physiological Measurements and Instrumentation Laboratory, Department of Instrumentation and Control Engineering, National Institute of Technology, Tiruchirappalli 620015, Tamil Nadu, India; 2 Department of Applied Electronics and Instrumentation Engineering, Rajagiri School of Engineering and Technology, Cochin 682039, Kerala, India

**Keywords:** encephalogram, epilepsy, seizures, machine learning, maximal overlap wavelet

## Abstract

In this work, an attempt has been made to develop an automated system for detecting electroclinical seizures such as tonic-clonic seizures, complex partial seizures, and electrographic seizures (EGSZ) using higher-order moments of scalp electroencephalography (EEG). The scalp EEGs of the publicly available Temple University database are utilized in this study. The higher-order moments, namely skewness and kurtosis, are extracted from the temporal, spectral, and maximal overlap wavelet distributions of EEG. The features are computed from overlapping and non-overlapping moving windowing functions. The results show that the wavelet and spectral skewness of EEG is higher in EGSZ than in other types. All the extracted features are found to have significant differences (*p* < 0.05), except for temporal kurtosis and skewness. A support vector machine with a radial basis kernel designed using maximal overlap wavelet skewness yields a maximum accuracy of 87%. In order to improve the performance, the Bayesian optimization technique is utilized to determine the suitable kernel parameters. The optimized model achieves the highest accuracy of 96% and an MCC of 91% in three-class classification. The study is found to be promising, and it could facilitate the rapid identification process of life-threatening seizures.

## 1. Introduction

Epilepsy is one of the most common and devastating neurological disorders that affect people of all ages [1]. It is characterized by repetitive seizures, which are the results of abnormal and excessive brain activity. Certain seizures that are frequently accompanied by stereotypical clinical manifestations are referred to as electroclinical seizures. Tonic-clonic seizures (TCSZ) and complex partial seizures are typical examples of electroclinical seizures. In the case of TCSZ, the patient suffers severely due to the stiffening and twitching of the muscles. This also sometimes leads to a sudden unexpected death in epilepsy (SUDEP) if the patient is unattended [2]. On the contrary, seizures that could be associated with clinical manifestations and do not involve any clinical changes are called electrographic (EGSZ) [3]. Generalized and focal non-specific seizures are examples of electrographic seizures.

Video EEG is considered a golden standard method for the identification of electroclinical seizures, and it is one of convenient procedures followed in the clinical settings. One of the difficulties of this technique is that it cannot be implemented outside the clinical settings. In addition, the evaluation of video EEG is a tiring and time-consuming task for neurologists. Furthermore, the evaluation is subjective, and there is also a short supply of neurologists. It is reported that the therapeutic decisions and clinical trials of TCSZ remained unresolved due to a lack of reliable evidence of clinical manifestations [4]. Therefore, this study aims to develop an automated system to identify seizure types within a short span of time after the onset using scalp EEG.

Several time, frequency, and time-frequency approaches have been developed for the detection of epileptic and normal EEG signals. Short-time Fourier transform measures such as spectral peaks, surface area, and energy have been explored for the differentiation of epileptic and normal EEG signals [5]. Numerous machine learning and deep learning algorithms have been experimented with for the detection of these abnormal events [6,7,8]. In recent studies, researchers have shown interest in developing an EEG signal-processing framework for identifying electroclinical seizures. Accelerometer-based wearable devices have been used to provide objective data about the occurrences of tonic-clonic seizures [9]. Several time and frequency features of intracranial EEG have been employed to predict impending tonic-clonic seizures [2].

Wavelet transform is a popular time-frequency analysis tool used to capture the transient variations present in the signals [10]. The Maximal overlap discrete wavelet transform (MODWT), is an advancement of the discrete wavelet transform that accounts for the issues associated with sample size. It is a scale-invariant, highly redundant, nonorthogonal transform. The redundancy of this advanced technique makes it easier to match the decomposed wavelet and scaling coefficients with the original time series at each level, allowing for a quick comparison of the series and its decomposition. In addition, the coefficients of MODWT for various scales are found to be uncorrelated in most of the cases, which helps in deriving useful statistical measures for the prediction problems [11,12].

Recent research shows that the statistical distribution of EEG provides useful information about underlying pathological conditions. The statistical measures, namely the mean, variance, skewness, and kurtosis of wavelet coefficients, are found to have significant differences between seizure onset and spread regions [13]. Variations in spectral kurtosis have been utilized as one of the major criteria for detecting high-frequency oscillations (HFOs) in epileptic EEG [14,15]. In order to overcome the tiring process of identifying spikes and HFOs, spectral skewness has also been shown to be useful in separating epileptic and non-epileptic channels [16]. However, the applicability of higher-order moments has not been explored for the detection of different seizure types. In particular, it is essential to investigate the usefulness of these higher-order moments extracted from time, frequency, and time-frequency domains. To the best of our knowledge, the effectiveness of these moments in different domains has not been explored in epilepsy research.

In this study, an attempt has been made to develop an automated system for the detection of seizure types such as tonic-clonic, complex partial, and electrographic seizures from the higher-order statistical measures of scalp EEG. The skewness and kurtosis are extracted from time, frequency, and maximal overlap wavelet distributions for different window sizes with and without overlap. The extracted features are used to design models based on a support vector machine with a radial basis kernel function. Further, the kernel parameters are optimized using a Bayesian algorithm for better performance.

## 2. Materials and Methods

### 2.1. Description of Data

This study uses Temple University Hospital (TUH) data corpus, which is publicly available for analysis [17]. The database contains the patient report, which includes the details of medical history, clinical manifestations, and medications consumed by the patients. In this work, we considered seizures with clinical symptoms, namely tonic-clonic seizures (TCSZ), complex partial seizures (CPSZ), and seizures without any clinical manifestations. The seizures without medical complications are said to be electrographic seizures (EGSZ), and they may be classified as either focal or generalized based on the involvement of cortex regions. Only epileptic seizures recorded at 400 Hz are considered in this work. The total numbers of seizure onset channels associated with TCSZ, CPSZ, and EGSZ are 83, 114, and 282 respectively.

The TUH EEG Corpus consists of more than 40 unique channels configurations [18]. These channels were optimized into temporal central parasagittal montage. This was done to reduce the dimensionality and to provide reasonable level of performance [19]. This study considers only the contacts involved in the seizure onset regions. All onset channels were identified as per the annotation guidelines [20].

### 2.2. Feature Extraction

Feature extraction is an important step in the analysis of biomedical signals. It helps in obtaining vital information from the signals. In this study, the higher-order moments, namely, skewness, and kurtosis are extracted from the time, frequency, and time-frequency domains of EEG signals. Short-Time Fourier Transform (STFT) has been utilized to compute the power spectrum and spectral asymmetry. The mathematical expression for STFT is given by:

(1) $$X\left(n,\omega \right)=\overset{\infty }{\underset{m=-\infty }{\sum }}x\left[m\right]w\left[n-m\right]e^{-j\omega n}$$

where $x\left[m\right]$ is a short-time part of the input signal $x\left[n\right]$ at time n, $w\left[n\right]$ is the window function, and $X\left(n,\omega \right)$ is time-frequency coefficients. The discrete STFT is defined as:

(2) $$X\left(n,l\right)=\left.X\left(n,\omega \right)\right|\omega =\frac{2\pi l}{N}$$

*N* is the number of discrete frequencies and l=0,1,2…N−1

The spectrogram in logarithmic scale is:

(3) $$S\left(n,l\right)=log{\left|X\left(n,k\right)\right|}^{2}$$

#### 2.2.1. Temporal Skewness and Kurtosis

In probability theory and statistics, skewness is a measure of the asymmetry of the probability distribution of a real-valued random variable. If the distribution is skewed to right side, then the value of skewness is said to be positive, whereas if the distribution is skewed to left side, the skew value will be negative.

The skewness (*SK_T_*) is defined by [21]:

(4) $$SK_{T}=\frac{E{\left(x-\mu \right)}^{3}}{\sigma^{3}}\;$$

where μ and σ represent the mean and standard deviation of the distribution.

Kurtosis is a metric that determines whether a data distribution is tailed or peaked, and it is a useful tool for detecting outliers. The kurtosis of a normal distribution is zero. Distributions with a high kurtosis have heavy tails, while distributions with a low kurtosis have light tails. The mathematical expression of kurtosis is given by:

(5) $$KU_{T}=\frac{E{\left(x-\mu \right)}^{4}}{\sigma^{4}}\;$$

#### 2.2.2. Spectral Skewness (*SS_S_*) and Kurtosis (*SKs*)

The spectral skewness measures the asymmetry of the spectral power distribution around its centroid. The mathematical expression of this measure is given by [22],

(6) $$SS_{S}=\frac{\sum_{k=b1}^{b2}\;{\left(f_{k}-\mu_{1}\right)}^{3}s_{k}}{{\left(\mu_{2}\right)}^{3}\sum_{k=b1}^{b2}s_{k}}$$

where *b_1_* and *b_2_* are band edges, *k* is frequency corresponding to bin *k*, $\mu_{1}$ is spectral centroid, $\mu_{2}$ is spectral spread, and $s_{k}$ is the spectral value at bin *k*.

Spectral kurtosis is a measure of the spectral flatness around its centroid. The mathematical expression is given by [22]:

(7) $$SKs=\frac{\sum_{k=b_{1}}^{b_{2}}{\left(f_{k}-\mu_{1}\right)}^{4}s_{k}}{{\left(\mu_{2}\right)}^{4}\sum_{k=b_{1}}^{b_{2}}s_{k}}$$

#### 2.2.3. Maximal Overlap Discrete Wavelet

Wavelet transform is one of the most powerful time-frequency approaches for exploring the non-stationary variations of signals. In wavelet technique, the signals are represented as a linear combination of shifted and dilated versions of the mother wavelet. It provides good time-frequency resolution. One of the merits of wavelet is that it provides optimal time and frequency localization at low frequencies (long window) and high frequencies (short window), respectively. Continuous wavelets are represented by multiplying the integral of a signal by its scaled and shifted version. The mathematical expression of wavelet transform is:

(8) $$CWT\left(a,b\right)=\frac{1}{\sqrt{a}}\overset{\infty }{\underset{-\infty }{\int }}x\left(t\right)\psi \left(\frac{t-b}{a}\right)dt$$

where *a* and *b* are scaling and shifting parameters, respectively. ψ is wavelet function. One of the most reliable and digital implementations of wavelet transform is discrete wavelet transform (*DWT*). This is implemented using an efficient Mallat algorithm. This method is based on a filter bank decomposition. It uses a set of high-pass and low-pass filters that compute detail coefficients (*D*_1_* − Dj*) and approximation coefficient (*Aj*), respectively, at decomposition level *j*. In general, approximate coefficients represent low frequencies and detailed coefficients denote high frequencies. The *DWT* that uses the power of two is very convenient and efficient for discrete signals such as EEG [23].

The Discrete Wavelet Transform (*DWT*) that uses power of two is very convenient and efficient for discrete signals like EEG. The expression for *DWT* is:

(9) $$DWT\left(j,k\right)=\frac{1}{\sqrt{2^{j}}}\overset{\infty }{\underset{-\infty }{\int }}x\left(t\right)\psi \left(\frac{t-k2^{j}}{2^{j}}\right)dt\left(9\right)$$

The scaling parameter is set to $2^{j}$ and shifting function is set to $2^{j}k$, where *k = −∞*… −2, −1, 0, 1, 2, … *∞* and *j* = 1, 2, … *∞*. *j* is number of levels, which is fixed to six in this study.

The analysis in this work makes use of the maximum overlap discrete wavelet transform (MODWT). Selecting the appropriate wavelet is essential for signal analysis because there are several wavelet families available for signal categorization. The mother wavelet is selected based on the experimenter’s convenience and needs depending on the sort of bio-signal to be studied. The most popular wavelet for *DWT* research and the one with the best detection rate, according to the literature, is Daubechies. It has been found that the shape and frequency characteristics of Daubechies 4 (db4) wavelet is similar to that of EEG signals manifested during the seizures [24]. Therefore, Daubechies wavelet of order of 4 (db4) is selected as a wavelet function. It suggests that the most used and effective wavelet for seizure detection is db4. The MODWT is a linear filtering operation that transforms a series into coefficients related to variations over a set of scales. The traditional *DWT* approach is a highly redundant and nonorthogonal transformation [25]. But MODWT retains down-sampled values at each level of the decomposition that would be otherwise discarded by the *DWT*. The mathematical expressions for computing MODWT coefficients are:

(10) $${\tilde{W}}_{j,t}=\overset{L_{j}-1}{\underset{l=0}{\sum }}h_{j,l}X_{t-l}$$

(11) $${\tilde{V}}_{j,t}=\overset{L_{j}-1}{\underset{l=0}{\sum }}g_{j,l}X_{t-l}$$

where $h_{j,l}$ and $g_{j,l}$ are the filter functions, *t = *0, …* N *− 1,* N* is the length of signal samples, and *l = *0, 1, …* L* − 1, and *L* is the width of the filter. The *j^th^* level of detail and approximation coefficients are:

(12) $${\tilde{D}}_{j,t}=\overset{L_{j}-1}{\underset{l=0}{\sum }}h_{j,l}W_{t+l}$$

(13) $${\tilde{A}}_{j,t}=\overset{L_{j}-1}{\underset{l=0}{\sum }}g_{j,l}V_{t+l}$$

The signal can be reconstructed using $X\left(n\right)=\overset{j}{\underset{l=0}{\sum }}{\tilde{D}}_{j}+{\tilde{A}}_{j}$

**Figure 1:**
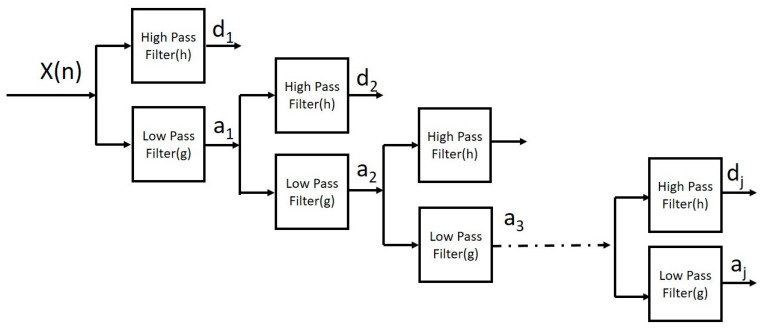
Overview of Wavelet decomposition. D_j_ is details at j^th^ level and a_j_ is approximation at j^th^ level.

Figure 1 depicts the *j^th^* level wavelet decomposition of signal. Applying the MODWT to a time series requires specifying parameters such as a wavelet filter and the level of decomposition. In this study, Daubechies wavelet filter is used for low-pass and high-pass filtering purposes, and the number of levels is fixed to six.

The signals are subjected to six-level decomposition, and the higher-order moments, namely skewness and kurtosis, are extracted from the coefficients. The Hann windowing function is employed to extract all the features in different domains. The features are computed from window lengths of 0.5 s and 1 s with and without overlapping. Overlap is designated as a percentage (0%) and 25% and 50 % of overlapping lengths are used in this study. The mathematical expressions of Hann windowing function [26]

(14) $$Hann\;W\left(n\right)=0.5\left[1-cos\left[2\pi \frac{n}{N+1}\right]\right]R_{N}\left(n\right)$$

where n=1,2,…N.

### 2.3. Support Vector Machine (SVM)

Support Vector Machine (*SVM*) is a supervised learning algorithm that separates two classes by creating an optimal decision boundary between them [27]. When the features of different classes have nonlinear relationship, then the linear *SVM* algorithm may not be a reliable choice. In this case, a kernel trick can be considered for the classification purpose. The kernel tricks will transform the data into higher dimensional space, where the classes can be linearly separated. In this study, a radial basis kernel is used to construct the decision boundary. Kernel equation of radial basis function is given by [28]:

(15) $$k\left(x,y\right)=\exp\left(-\frac{\|x-{y\|}^{2}}{2\sigma^{2}}\right)$$

(16) $$kernel\;scale\left(\gamma \right)=\frac{1}{2\sigma^{2}}$$

where x−y is distance between *x* and *y*.

### 2.4. Bayesian Optimization

The purpose of Bayesian algorithm is to provide optimal solution for the objective functions. It is widely utilized in the field of cutting-edge artificial intelligence to tune the hyper parameters. It has been reported that the technique clearly outperformed the genetic algorithm, particle swarm optimization, and other algorithms [29]. Recently, it has been applied to tune the hyper parameters of machine learning algorithm [30]. It uses Gaussian process and acquisition function for the optimization task. In each iteration, the surrogate model was updated, and acquisition function finds the next potential point to evaluate the objective function. The expression for the objective function of SVM [31] is:

(17) $$min_{\omega ,\xi }=\left\{\frac{1}{2}\|{\omega \|}^{2}+C\underset{i}{\sum }\xi_{i}\right\}$$

where ω represents the hyperplane vector, and *C* is the weight of the penalty function, which is defined by the sum of all ξ slack variables. This process will continue until the global minimum is reached.

Two important parameters of RBF-SVM classifier are kernel scale and box constraint. Kernel scale (ℽ) specifies the shape of the peak, and box constraint (*C*) controls the tradeoff between maximization and errors of training data. In this research, Bayesian optimization technique is employed to enhance the performance of classification tasks by fixing the suitable values for kernel scale and C.

The performance of the model is analyzed using the following metrics, namely, sensitivity (*SN*), specificity (*SP*), precision (*PR*), accuracy (*Ac*), and F1-score (*F1*). The mathematical representation is given below.

(18) $$SN=\frac{TP}{TP+FN}$$

(19) $$SP=\frac{TN}{TN+FP}\;$$

(20) $$PR=\frac{TP}{TP+FP}\;$$

(21) $$Ac=\frac{TP+TN}{TP+TN+FP+FN}\;$$

(22) $$F1=\frac{2TP}{2TP+FP+FN}\;$$

where *TP* = True Positive, *TN*= True Negative, *FP* = False Positive, and *FN* = False Negative.

Although measures such as accuracy and F1 score are popular in the evaluation of classification models, the Matthews correlation coefficient (*MCC*) is one of the more reliable measures for both binary and multi class classification [32,33]. *MCC* only yields a high score if all four parameters have high scores. In other words, if any value in the confusion matrix goes down, the *MCC* score also drops, whereas F1 score is insensitive to *TP* and highly sensitive to FN [33]. Therefore, in order to evaluate the model performance, *MCC* is also incorporated in this research. The mathematical expression of *MCC* is written as:

(23) $$MCC=\frac{TP\times TN-FP\times FN}{\sqrt{\left(TP+FP\right)\left(TP+FN\right)\left(TN+FP\right)\left(TN+FN\right)\;}}$$

**Figure 2:**
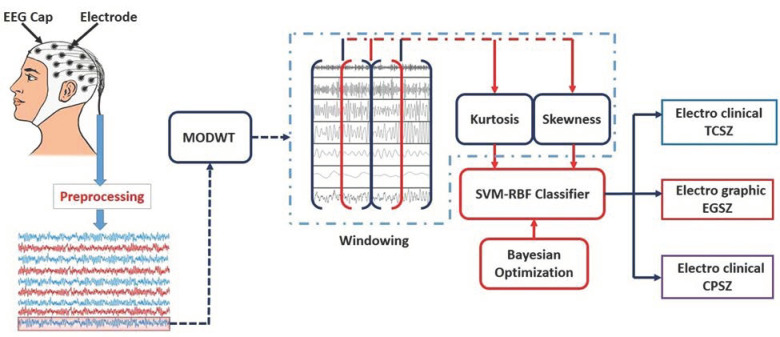
Block diagram representation of proposed system.

Figure 2 illustrates the proposed seizure type detection system that uses the higher-order moments from maximal overlap wavelet distribution.

## 3. Results

**Figure 3:**
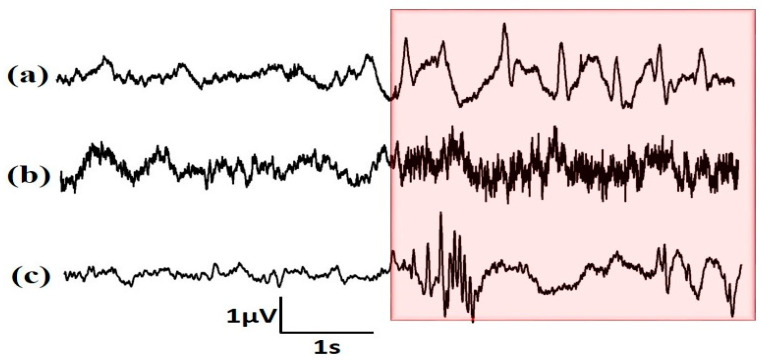
Representative EEG channel of (**a**) EGSZ, (**b**) CPSZ and (**c**) TCSZ. The electrical activities of brain from onset are highlighted in the box.

The main aim of this work is to develop a scalp EEG based automated system for the classification of TCSZ, CPSZ, and EGSZ. The first four seconds of onset are considered with an intention of predicting seizures early from the onset. The representative signals of EGSZ, CPSZ, and TCSZ are shown Figure 3. The box represents the electrical activities of the brain from the seizure onset that are considered for the analysis.

**Figure 4:**
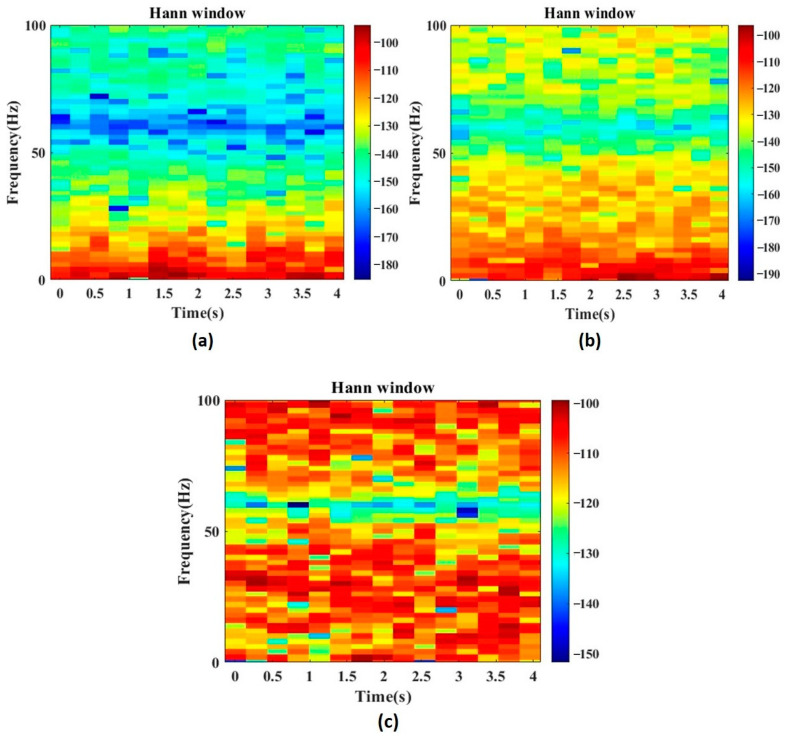
Spectrogram of (**a**) EGSZ, (**b**) CPSZ and (**c**) TCSZ. Hann window function is used to extract power spectrum of the signals. Colors indicate the intensity of power component with respect to frequency.

**Figure 5:**
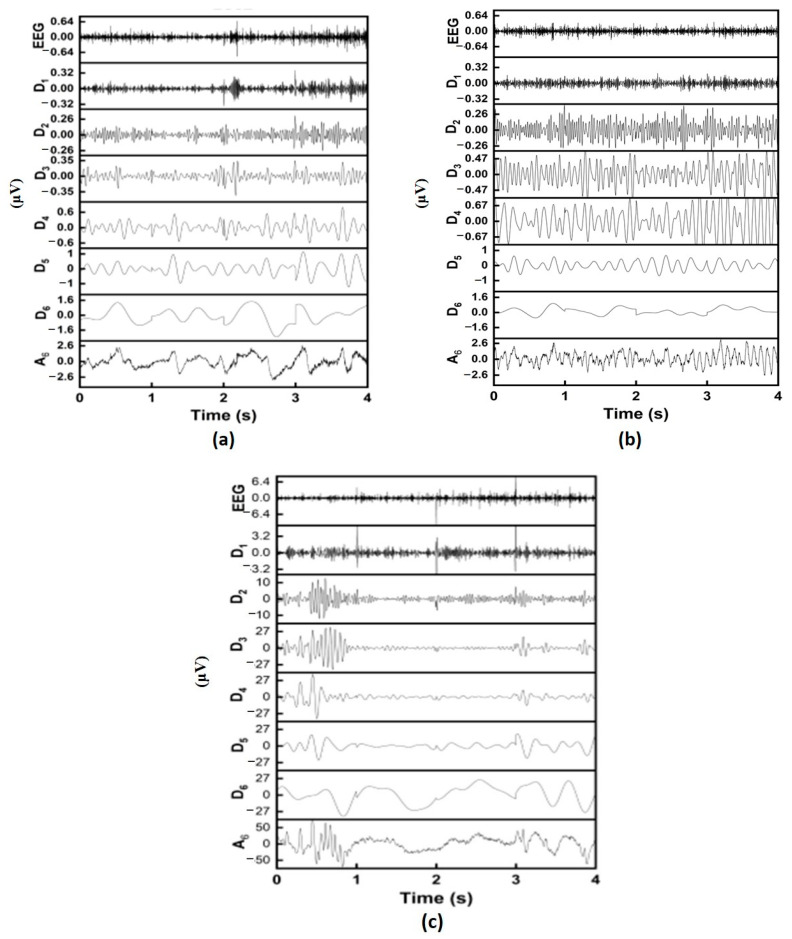
Maximal overlap discrete wavelet transform of representative signals. (**a**) EGSZ, (**b**) CPSZ, and (**c**) TCSZ.

Figure 4 depicts the spectrogram of the representative signals of TCSZ, CPSZ and EGSZ. It is apparent from the spectrograms that the power of the signal at lower frequency range is more in all the seizures. However, in TCSZ, a large amount of power is distributed even in the high frequency regions. Figure 5 represents the MODWT decomposition of representative signal of TCSZ, CPSZ and EGSZ. The sixth level decomposition yields seven different frequency bands that include six detailed coefficients and one approximate co-efficient (*D*_1_, *D*_2_, *D*_3_, *D*_4_, *D*_5_, *D*_6_, and *A*_6_). The frequency components associated with *D*_1_, *D*_2_, *D*_3_, *D*_4_, *D*_5_, *D*_6_ and *A*_6_ are 100–200 Hz, 50–100 Hz, 25–50 Hz, 12.5–25 Hz, 6.25–12.5 Hz, 3.12–6.25, and 0–3.12 Hz, respectively.

**Figure 6:**
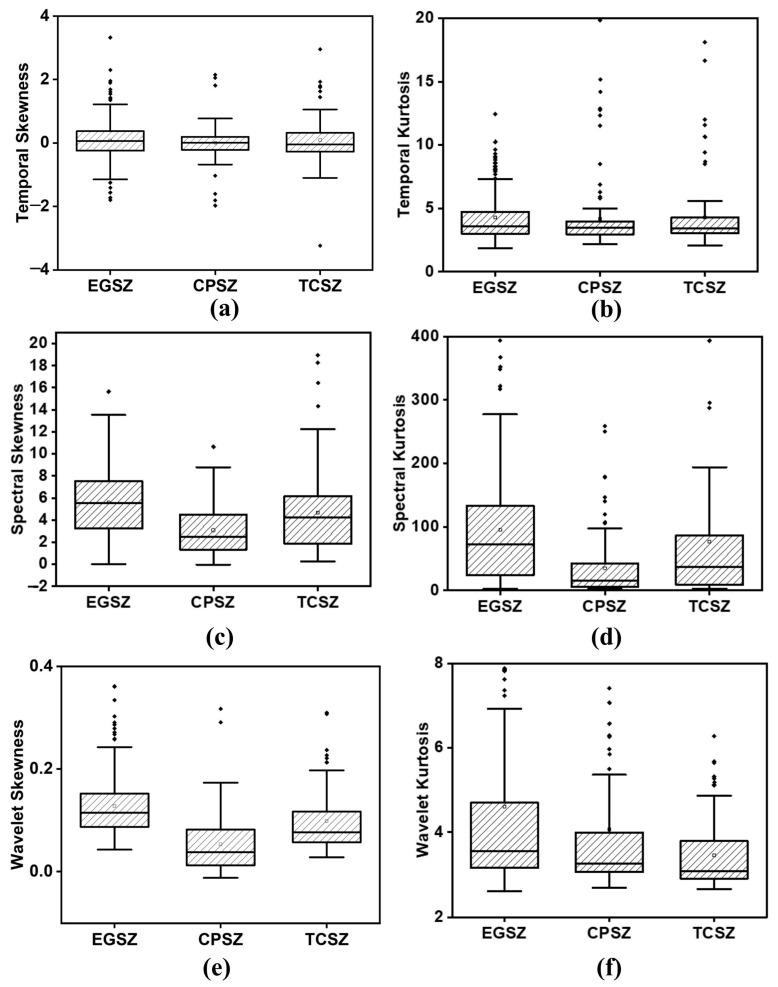
Distribution of (**a**) temporal skewness, (**b**) temporal kurtosis (**c**) spectral skewness (**d**) spectral kurtosis (**e**) wavelet skewness and (**f**) wavelet kurtosis. Window function of 0.5 s with an overlap of 50%.

Figure 6 presents the distribution of temporal, spectral, and wavelet skewness and kurtosis extracted from the window length of 0.5 s with 50% overlap. The box plot shows the distribution of average skewness and kurtosis computed from each EEG channel. The median of skewness and kurtosis are found to be higher in EGSZ than TCSZ and CPSZ in spectral and wavelet domain. In the case of temporal skewness and kurtosis, only a very little difference can be seen in the median values among seizure types. It is also noticed that the percentage of overlap in temporal measures is higher than in spectral and wavelet measures. The results of the ANOVA test show that all the extracted features except the temporal kurtosis and skewness are found to have significant difference between the seizure types (*p* < 0.05). The number of EEG channels in the different seizures seemed to be highly imbalanced, and therefore, the development of detection models will lead to unreliable performance. There are several solutions for handling the class imbalances. These include techniques such as undersampling and oversampling. In the undersampling technique, we randomly select a number of samples from the majority class, whereas oversampling entails creating artificial examples of the minority class. The synthetic minority oversampling technique and adaptive synthetic algorithms are examples of oversampling [34].

In this study, random undersampling (RUS) technique was adapted to handle the class imbalance. RUS approach selects 83 samples at random from the majority class and ensures that the data balance is maintained for training and testing. The developed model was evaluated using 10-fold cross-validation. Table 1 compares the performance of temporal, spectral and wavelet measures for different window sizes and overlapping lengths. It can be observed that wavelet and spectral skewness performed better than others. In particular, the wavelet skewness from the 500 ms window with 50% overlap yielded the highest accuracy of 87% in detecting these three seizure types. Beside this, the model based on spectral skewness achieved a maximum accuracy of 85.9%. The performance of temporal skewness and kurtosis is lower when compared to spectral and wavelet models. To increase the performance of the models further, the hyper parameters of RBF-SVM are optimized using the Bayesian approach. For the optimization, we considered only the feature sets computed from a 500 ms window length with 50% overlap, which could provide maximum performance.

**Figure 7:**
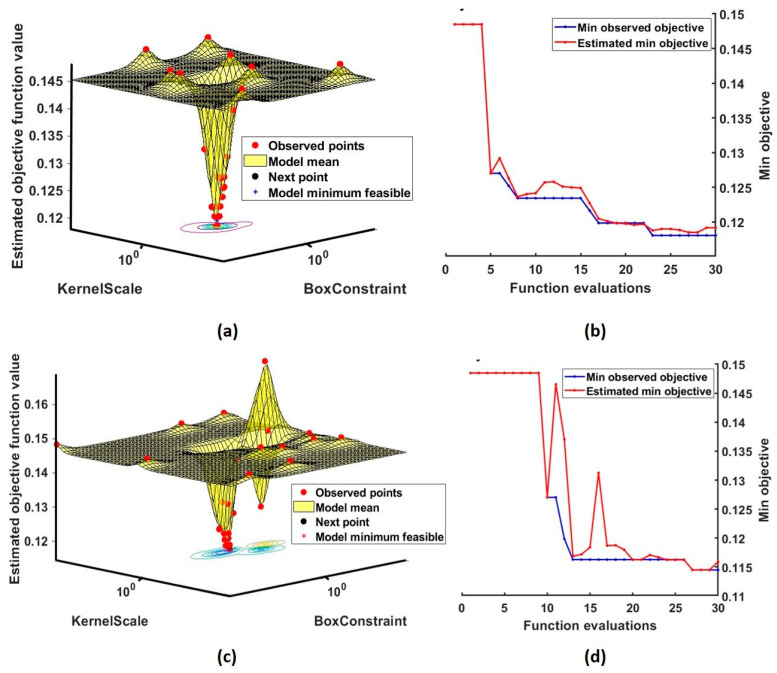
Bayesian optimization of hyperparameters of RBF-SVM. (**a**,**b**) represent the optimization process of wavelet skewness and (**c**,**d**) represents the minimization process of wavelet kurtosis. The objective function values, kernel parameters in 30 evaluations are shown.

Table 2 lists the optimized parameter values, along with the accuracy. It was found that the temporal skewness and kurtosis obtain the best feasible points when the parameters (C, ℽ) were at (6.3, 10.4) and (2.43, 7.65) respectively. For temporal skewness, it took 124 milliseconds to reach the best minimum objective function value of 0.1192. Temporal kurtosis model could obtain the minimum function value of 0.14132 in 138 milliseconds. Similarly, the model based on spectral skewness has obtained the feasible objective function value of 0.0141. At this point, the hyperparameter values were 6.86 for C and 7.18 for ℽ. The time taken to complete the optimization process was 138 milliseconds. For the model based on spectral kurtosis, the minimum value of objective function is 0.134 and its hyperparameters were 1.5 (C) and 2.74 (ℽ). Figure 7 depicts the optimization process of models based on the wavelet skewness and kurtosis in 30 evaluations. From these results, it can be observed that the box constraint and kernel scale values of wavelet skewness are lower than the skewness from temporal and spectral domain. It can be seen that the wavelet skewness yields a maximum accuracy of around 96% for C = 2.84 and **ℽ =** 6.34. Overall, the detection rate increased by about a minimum of 9% after optimization. It was also found that the performances of wavelet kurtosis and spectral skewness and kurtosis were considerably improved due to the Bayesian optimization.

The metrics of the improved models are shown in Table 3. The performances of wavelet and spectral skewness are similar. However, the maximum SP, F1, and MCC values are observed in the model designed using wavelet skewness. The results show that the model can detect the three seizure types with the SN of 94.7%, SP of 97.3%, PR of 94.8%, F1- score of 94.7%, and MCC of 91.4%. The highest MCC values show the effectiveness of wavelet skewness in the automated recognition of epileptic seizure types. It is also important to note that the accuracy of the wavelet kurtosis increased by about 10% after optimization. There were also some notable improvements in the temporal skewness-based classification models. However, the detection rate was not as similar to the other measures. The maximum MCC found in temporal skewness was only 74, whereas in the case of wavelet skewness, the model achieved 91.

## 4. Discussion

**Figure 8:**
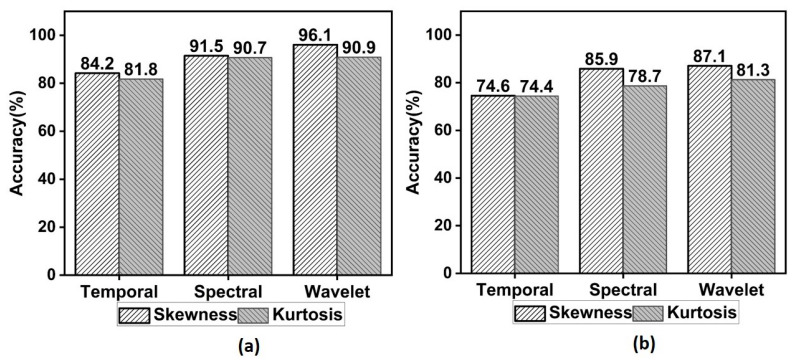
Barchart of detection rate (%) in temporal, spectral and wavelet domains (**a**) after optimization (**b**) before optimization.

In this work, maximal overlap wavelet transform of higher-order moments is proposed to differentiate the TCSZ, CPSZ, and EGSZ from scalp EEG. The effectiveness of the proposed measure is evaluated for Hann window functions. Wavelet domain features yield maximum performance compared to temporal and spectral domain features. It is also found that 50% of overlap with 0.5 s data perform the maximum in all domains. There are several wavelet families available for signal characterization, and choosing the right wavelet is crucial for signal analysis. The Daubechies family of wavelets with order 2 or 4, which offered better results for biosignal characterization, has been used mostly in the research work [24]. Decomposition levels also play an important role in optimization process [35]. However, in this study, it is observed that there is no significant change in the classification accuracy after the 6th level of decomposition. Further, the impact of optimizing the kernel parameters is also investigated in this research. The detection rate before and after the optimization is presented in Figure 8.

Several studies on epilepsy mainly focused on discriminating seizure and non-seizure events over the past decades. There are only a few studies that have made an attempt to develop algorithms for the detection of seizure types. Table 4 provides a brief comparison of our proposed work with the models based on classical learning algorithms reported in the literature. Wavelet decomposition methods were widely used for the detection of seizure types. Three features, namely fuzzy entropy, logarithmic of the squared norm, and fractal dimension, were computed from the wavelet coefficients of EEG signals. These coefficients were decomposed by two-band energy localized orthogonal wavelet filter bank. These features, along with the quadratic SVM model, yielded a maximum accuracy of 79.34% and an F1 score of 88% [36]. In another study, features such as root mean square value, variance, standard deviation, log entropy, and maximum frequency were extracted from coefficients and were used to develop models based on bagged tree and k−NN. The maximum accuracy of 82% was achieved with the bagged tree algorithm [37]. The energy of the wavelet coefficients was also exploited for the characterization of EEG signals during TCSZ and EGSZ. The classification accuracy of 87% was achieved with the k−NN model based on these measures [38]. Recently, the SVM-polynomial kernel-based learning model with the entropy from the seven scale wavelet coefficients was shown to be useful in detecting the TCSZ with a maximum accuracy of 95% [39].

In this work, machine learning models based on higher-order moments from three domains and RBF-SVM are proposed to differentiate three types of seizures, namely TCSZ, CPSZ, and EGSZ from scalp EEG. The impact of optimizing kernel parameters is also investigated in this research. It is observed that wavelet skewness performs better than others. The results show that the proposed system can differentiate the TCSZ, CPSZ, and EGSZ with an average accuracy and MCC of 96% and 91.4%, respectively. It is important to note that the proposed system utilizes only four seconds of signals from the seizure onset for the detection of seizures. The computational load for the calculation of wavelet skewness is only 2.3 milliseconds per channel. Therefore, the proposed system could be used either in clinical settings or at home to detect the seizures with and without clinical manifestations. The model is developed using MATLAB R 2019b and implemented on an Intel^®^ Core™ i7 8550U with the clock frequency of 1.80 GHz. The study considers relatively a low number of epileptic channels for the development and testing of automated seizure detection system. This limitation will be overcome by incorporating a greater number of seizures in the future.

## 5. Conclusions

In this research, a system is proposed to differentiate the TCSZ, CPSZ and EGSZ from the scalp EEG. For this purpose, the EEG signals from Temple University and Hospital database are considered for the analysis. Recent studies have shown that higher-order moments provide valuable information about pathological conditions. Therefore, in this study, an attempt has been made to analyze the applicability of the skewness and kurtosis from three different signal processing domains in the detection of TCSZ, CPSZ, and EGSZ. The variations of skewness and kurtosis in time, frequency, and time-frequency domains are analyzed for varied window sizes with and without overlap. Further, these features are used to design a model based on SVM-RBF for classification purposes. Then, the random undersampling technique is implemented to avoid issues related to class imbalance. The results show that the wavelet and spectral measures are higher in EGSZ than TCSZ and CPSZ. The maximum accuracy of 87% is achieved from the model based on wavelet skewness. In order to improve the performance, the Bayesian optimization technique is adapted to find suitable kernel parameters of RBF. The performance of the model is improved by around 9% after optimization. The highest accuracy of 96%, F1-measure of 94.7%, and precision of 94.8% are obtained by the wavelet skewness for the three-class classification. Therefore, it appears that the proposed model has the potential to detect seizure types and improve the efforts of therapeutic decisions. The proposed seizure prediction framework can also be extendable for applications related to wearable sensors.

## Figures and Tables

**Table 1:** Accuracy of detection models.

	Skewness						Kurtosis					
Window size	0.5 s			1 s			0.5 s			1 s		
Overlap (%)	0	25	50	0	25	50	0	25	50	0	25	50
Temporal domain	73.5	74.5	74.6	68.3	70.5	61.1	71.3	69.4	74.4	67.2	67.2	75
Spectral domain	83.6	85.0	85.9	81.4	84.9	84.3	77.7	74.4	78.7	75	76.7	77.3
Wavelet domain	85.3	83.2	87.1	82.1	81.7	81.0	79.4	80.7	81.3	80.2	78.5	79.3

**Table 2:** Average accuracy after optimization of RBF-SVM.

	Skewness			Kurtosis		
Method	Box Constraint (C)	Kernel Scale (ℽ)	Accuracy (%)	Box Constraint (C)	Kernel Scale (ℽ)	Accuracy (%)
Temporal domain	6.3	10.4	84.22	2.43	7.65	81.83
Spectral domain	6.86	7.18	91.53	1.5	2.74	90.76
Wavelet domain	2.84	6.34	96.12	1.731	5.6	90.96

**Table 3:** Performance metrics of RBF SVM after the optimization.

	Skewness (%)					Kurtosis (%)				
Method	SN	SP	PR	F1	MCC	SN	SP	PR	F1	MCC
Temporal	87.6	85.8	85.5	86.5	74.2	83.1	81.9	82.1	82.6	71.7
Spectral	95.3	93.9	93.1	93.2	90.1	92.5	89.5	89.1	90.8	85.6
Wavelet	94.7	97.3	94.8	94.7	91.4	92.7	96.3	92.6	92.6	90.0

**Table 4:** Comparison of classical machine learning based seizure type detection methods.

Authors	Method + Classifiers	Performance
Sharma et al. [36]	Wavelet filter banks + SVM	Ac-79.34%; F1 = 88%
Niamh M et al. [37]	Wavelet + k-NN + bagged tree	Ac = 82%
M. Joseph et. al. [38]	Wavelet energy + k-NN	Ac = 87.6%
M. Joseph et al. [39]	Wavelet entropy + SVM	Ac = 95.5%, F1 = 95.9%
Proposed	Spectral skewness + SVM	Ac = 95.18%; F1 = 95.24%; MCC = 0.90%

## Data Availability

https://isip.piconepress.com/projects/tuh_eeg/html/downloads.shtml (accessed on 17 March 2021).
